# Prognostic and clinicopathological value of prognostic nutritional index in patients with multiple myeloma: a meta-analysis

**DOI:** 10.3389/fonc.2025.1545096

**Published:** 2025-09-19

**Authors:** Yanchen Nie, Zongxin Zhang, Xiaohuan Tang

**Affiliations:** ^1^ Department of Hematology, Huzhou Transportation Hospital, Huzhou, Zhejiang, China; ^2^ Clinical Laboratory, Huzhou Central Hospital, Affiliated Central Hospital of Huzhou University, Huzhou, Zhejiang, China; ^3^ Clinical Laboratory, The Third People’s Hospital of Deqing, Deqing Hospital of Hangzhou Normal University, Huzhou, Zhejiang, China

**Keywords:** prognostic nutritional index, multiple myeloma, meta-analysis, prognosis, biomarker

## Abstract

**Background:**

Prognostic nutritional index (PNI) has been extensively investigated for its effect on forecasting multiple myeloma (MM) survival; however, the conclusions are conflicting. This meta-analysis identified an accurate MM prognosis forecasting role for the PNI.

**Methods:**

We systematically searched PubMed, Web of Science, Embase, Cochrane Library, and CNKI databases until July 2, 2025, and evaluated the overall survival (OS) and progression-free survival (PFS) forecasting ability of the PNI by determining pooled hazard ratios (HRs) with 95% confidence intervals (CIs).

**Results:**

This study included seven articles involving 1120 participants. From the pooled findings, a lower PNI exhibited a remarkable correlation with unfavorable OS (HR = 2.62, 95% CI = 1.76–3.89) and shorter PFS (HR = 1.52, 95% CI = 1.23–1.89, p<0.001) of MM. Additionally, lower PNI was significantly associated with ISS stage III (odds ratio [OR]=1.80, 95% CI = 1.19–2.73, p=0.005). However, PNI did not have a marked correlation with sex (OR = 1.02, 95% CI = 0.71–1.47, p=0.900), age (OR = 1.1, 95% CI = 0.70–1.93, p=0.558), and lactate dehydrogenase (OR = 0.98, 95% CI = 0.57–1.69, p=0.955) in MM. The meta-analysis had some limitations, such as retrospective design, small sample size, and inconsistent cut-off values of PNI.

**Conclusion:**

Collectively, the present work including 1120 patients showed the relationship between a lower PNI and unfavorable MM OS and PFS. Furthermore, a lower PNI was significantly associated with an advanced ISS stage of MM. The PNI can be a creditable and cost-effective factor for forecasting MM prognosis.

## Introduction

Multiple myeloma (MM) accounts for the bone marrow plasma cell cancer, occupying approximately 10% of hematologic malignancies (1). It is the second most prevalent blood cancer in adults, with an average age at diagnosis of 65 years (2). The incidence rate of MM is 1% among all tumors, affecting 4.5 – 6 individuals per 100,000 population (3). MM develops slowly and lacks clear symptoms initially, making it prone to misdiagnosis in clinical settings (4). Despite improvements in treatment, many patients with MM experience relapse and develop resistance to standard therapies, resulting in the disease being mostly incurable (5, 6). Identifying high-risk factors using risk stratification among MM cases can significantly help in planning treatment and assessing patient prognostic outcomes (7). Consequently, identification of novel and reliable markers for MM prognosis is important.

Current research highlights that nutritional status, which is subject to change, is a crucial element in cancer prognosis (8). Prognostic models based on data from physical examinations and laboratory results have been established to predict tumor progression and patient prognosis. The prognostic nutritional index (PNI) can comprehensively determine nutrition and immunity and was initially created to assess preoperative nutritional and immune conditions in patients with gastrointestinal cancer (9). PNI is computed as follows; PNI = 10 × serum albumin (ALB) level (g/dL) + 0.005 × lymphocyte quantity (number/mm³) (9). A low PNI is widely suggested to be of significant prognostic significance for different cancers, such as osteosarcoma (10), prostate cancer (11), renal cell carcinoma (12), lung cancer (13), and hepatocellular carcinoma (14). Previously, the prognostic value of PNI for MM has been analyzed; however, the findings are inconsistent (15–21). PNI has been shown to significantly predict MM prognosis (16–19). However, other studies have suggested no obvious association between them (15, 20). Therefore, we performed this meta-analysis to determine the precise prognostic effect of the PNI on MM. The correlations between PNI and MM clinicopathological features were investigated in this meta-analysis.

## Materials and methods

### Study guideline

This work was completed based on the Preferred Reporting Items for Systematic Reviews and Meta-Analyses guidelines (22).

### Ethics statement

Experiments on humans or animals were not performed in this study, which waived ethical approval.

### Literature search

We systemically searched PubMed, Web of Science, Embase, Cochrane Library, and CNKI until July 2, 2025, using keywords, such as prognostic nutritional index (PNI) and multiple myeloma or myeloma. No language restrictions were applied. References were manually retrieved to identify any literature that might have been missed in the initial search.

### Study selection

The following studies were enrolled; (1) MM diagnosis based on pathology, (2) patients with MM were treated by any methods in adherence with the treatment guidelines, (3) PNI was derived before treatment, based on ALB level and lymphocyte counts. And ALB and lymphocyte counts were calculated at one time, (4) studies investigating the relationship between PNI and MM survival, (5) obtainable or computable hazard ratios (HRs) with 95% confidence intervals (CIs), (6) available PNI threshold, and (7) unrestricted language. Moreover, these studies excluded (1) reviews, case reports, meeting abstracts, letters, and comments, (2) studies that enrolled duplicate patients, and (3) animal studies.

### Data acquisition and quality evaluation

Two researchers (Y.N. and Z.Z.) acquired the data from these studies. Disputes between them were settled by a third researcher (X.T.). We obtained information on first author’s name, country, year, sample size, sex, study period, age, study design, study center, International Staging System (ISS) stage, treatment, threshold PNI, threshold determination method, survival outcomes, survival analysis, follow-up, HRs, and 95% CIs. OS and PFS was considered the primary and secondary outcomes, respectively. We used the Newcastle–Ottawa Quality Assessment Scale (NOS) to assess the enrolled study quality (23), and studies with 7 – 9 points were of high quality.

### Statistical analysis

We computed HRs with 95% CIs to assess the MM OS and PFS forecasting abilities of the PNI. Among-study heterogeneity was assessed using Cochran’s test and I^2^ statistics. I^2^ > 50% or p < 0.10 (Q-test) suggested distinct heterogeneity; therefore, a random-effects model must be used; otherwise, a fixed-effects model would be adopted. Subgroup analyses were conducted to investigate the prognostic value of the PNI in patient groups. The relationships between PNI and MM clinicopathological characteristics were explored using combined ORs with 95% CIs. Stability and creditability were assessed through sensitivity analysis, which involved eliminating every individual article and recalculating the HRs and 95% CIs. Possible publication bias was evaluated using funnel plot, Begg’s test, and Egger’s test. We employed Stata 12.0 software (Stata Corp., College Station, Texas, USA) for statistical analyses. Statistical significance was set at p<0.05.

## Results

### Literature screening

**Figure 1:**
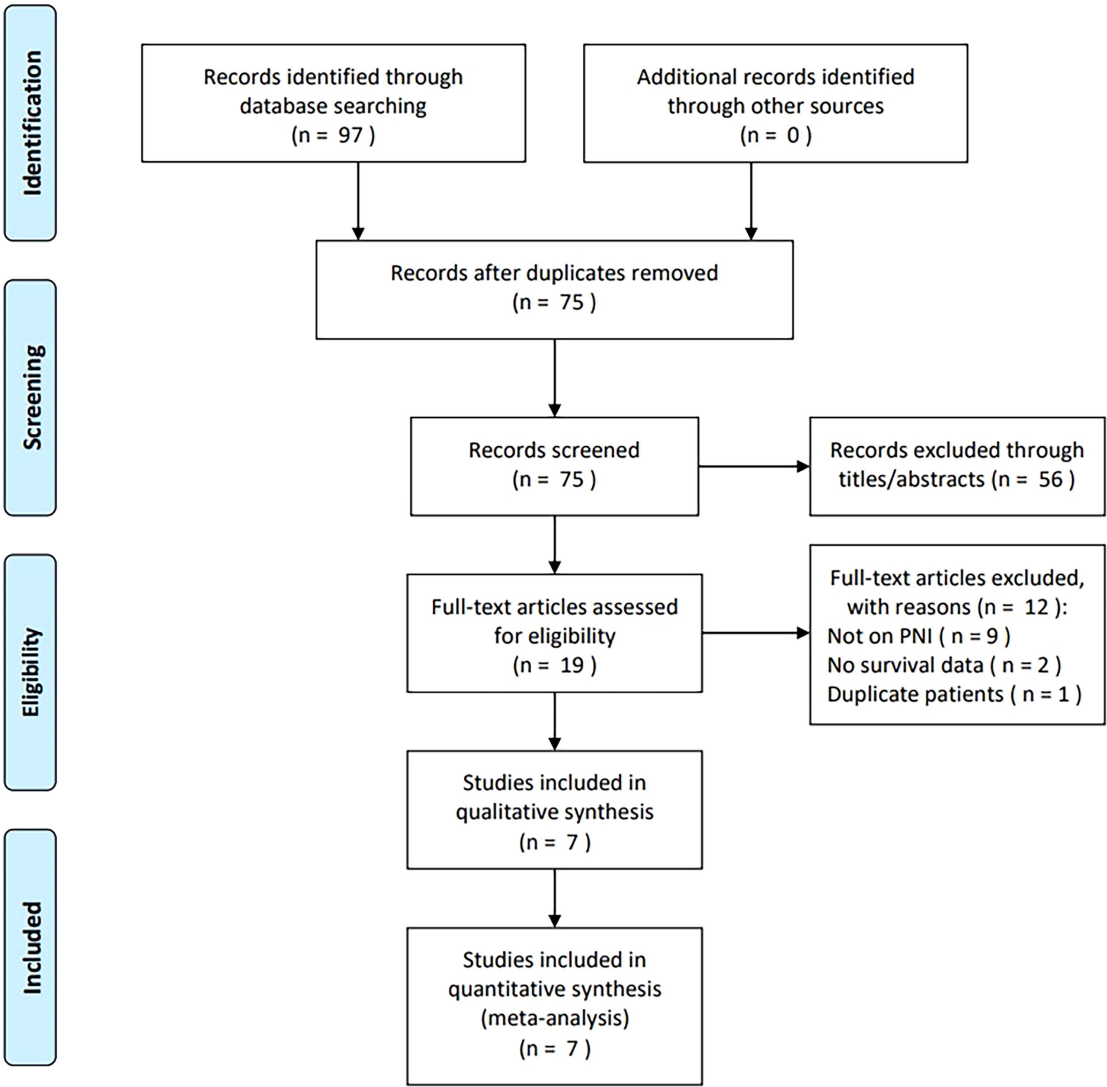
Study selection flow diagram.

Ninety-seven studies were obtained from the primary search, of which 75 were retained after removing duplicates (**Figure 1**). We removed 56 articles after reading the title and abstract owing to irrelevance or animal studies. We assessed 19 articles through full-text reading, of which 12 were excluded because they were not on PNI (n=9), survival information was unavailable (n=2), and repeated participants were enrolled (n=1). Therefore, we enrolled seven studies with 1120 patients (15–21) in the meta-analysis (**Figure 1**).

### Enrolled article features

The retrospective fundamental article features (15–21) published between 2020 and 2024 are shown in **Table 1**. Six of them were conducted in China (16–21) and one in Germany (15). The sample size was 74 – 243 (median; 160). Six studies were published in Chinese (16–21) and one in English (15). Six studies enrolled MM cases of ISS stage I–III (15–19, 21) and one recruited relapsed/refractory (R/R) patient with MM (20). One study treated patients with autologous stem cell transplantation (ASCT) (15) and six studies applied chemotherapy (16–21). The chemotherapy regimens include Bortezomib + Dexamethasone, Bortezomib + Cyclophosphamide + Dexamethasone, and VAD ± T (18, 21). All included studies recorded the PNI value before treatment. The PNI thresholds ranged from 39.8 – 47.2 (median; 44.45). Four studies determined the threshold using the receiver operating characteristic (ROC) curve (16, 19–21) and one each applied literature (15), X-tile software (17), and the median value (18) Six articles mentioned PNI significance in predicting OS (15, 16, 18–21) and four mentioned the relationship between PNI and PFS (15, 17, 20, 21) in MM. Four articles derived HRs and 95% CIs on univariate regression (17, 19–21), whereas three applied multivariate regression (15, 16, 18). Our studies had NOS scores of 7 – 9 (median; 8), suggesting a high quality (**Table 1**). The detailed NOS scores of each included study were shown in **Supplementary File 1**.

**Table 1:** Basic characteristics of studies included in this meta-analysis.

Study	Year	Country	Sample size	Gender (M/F)	Age (years) Median(range)	Study period	Study center	ISS stage	Treatment	PNI cut-off value	Cut-off determination	Survival outcomes	Survival analysis	Follow-up (months) Median(range)	NOS score
Witte, H. M. (15)	2020	Germany	224	130/94	59(35 - 76)	2010-2018	Multicenter	I-III	ACST	45	Literature	OS, PFS	Multivariate	58(1 - 248)	9
Liang, F. (16)	2021	China	157	88/69	64(30 - 91)	2014-2018	Single center	I-III	Chemotherapy	44.45	ROC curve	OS	Multivariate	24(0.5 - 71.0)	8
Chen, B. R. (17)	2022	China	160	95/65	63	2015-2018	Single center	I-III	Chemotherapy	43.4	X-tile	PFS	Univariate	1-60	8
Chen, X. S. (18)	2023	China	243	138/105	66(42 - 92)	2016-2022	Single center	I-III	Chemotherapy	47.2	Median value	OS	Multivariate	1-92	8
Li, Q. F. (19)	2024	China	74	46/28	68(65 - 80)	2020-2022	Single center	I-III	Chemotherapy	39.8	ROC curve	OS	Univariate	7(1 - 17)	7
Liu, J. (20)	2024	China	101	49/52	≤60y: 30 >60y: 71	2016-2023	Single center	R/R	Chemotherapy	41.9	ROC curve	OS, PFS	Univariate	1-130	8
Wang, L. (21)	2024	China	161	93/68	63(31 - 84)	2015-2022	Single center	I-III	Chemotherapy	45	ROC curve	OS, PFS	Univariate	35.4	7

R/R, relapsed/refractory; M, male; F, female; ISS, International Staging System; ASCT, autologous stem cell transplantation; ROC, receiver operating characteristic; OS, overall survival; PFS, progression-free survival; PNI, prognostic nutritional index; NOS, Newcastle-Ottawa Scale.

### PNI and OS

**Figure 2:**
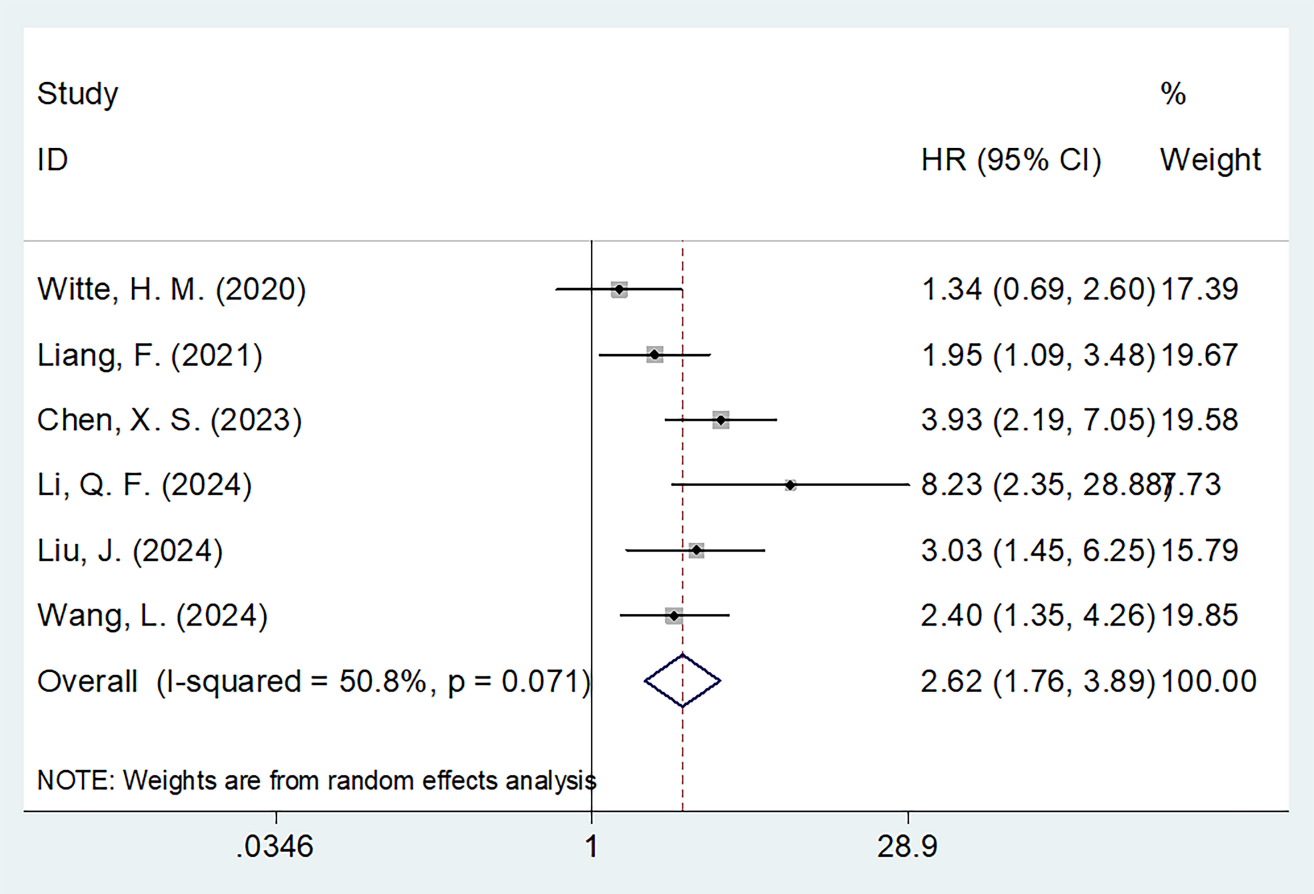
Forest plots of the association between PNI and OS in patients with MM.

Six studies with 960 patients (15, 16, 18–21) mentioned the role of PNI in predicting OS. A random effects model was used because of obvious heterogeneity (I^2^ = 50.8%, p=0.071). Based on the pooled results, decreased PNI was significantly correlated with dismal OS in MM (HR = 2.62, 95% CI = 1.76–3.89, p<0.001; **Figure 2**; **Table 2**). Subgroup analyses revealed that a significant effect of PNI on forecasting OS is not influenced by sample size, ISS stage, cut-off value, or survival analysis type (**Table 2**). Additionally, the PNI remarkably forecast dismal OS in subgroups of studies in China, patients receiving chemotherapy, and threshold determination based on the ROC curve (p<0.05; **Table 2**).

**Table 2:** Subgroup analysis of the prognostic role of PNI for OS in patients with MM.

Subgroups	No. of studies	No. of patients	Effects model	HR (95%CI)	p	Heterogeneity I2(%) Ph	
Total	6	960	Random	2.62(1.76 - 3.89)	<0.001	50.8	0.071
Country							
Germany	1	224	–	1.34(0.69 - 2.60)	0.391	–	–
China	5	736	Fixed	2.87(2.14 - 3.86)	<0.001	32.5	0.205
Sample size							
≤160	3	332	Random	3.06(1.55 - 6.06)	0.001	53.9	0.114
>160	3	628	Random	2.37(1.31 - 4.28)	0.004	65.0	0.058
ISS stage							
I-III	5	859	Random	2.57(1.60 - 4.14)	<0.001	69.6	0.042
R/R	1	101	–	3.03(1.46 - 6.29)	0.003	–	–
Treatment							
ASCT	1	224	–	1.34(0.69 - 2.60)	0.391	–	
Chemotherapy	5	736	Fixed	2.87(2.14 - 3.86)	<0.001	32.5	0.205
PNI cut-off value							
<45	3	332	Random	3.06(1.55 - 6.06)	0.001	53.9	0.114
≥45	3	628	Random	2.37(1.31 - 4.28)	0.004	65.0	0.058
Cut-off determination							
ROC curve	4	493	Fixed	2.58(1.83 - 3.63)	<0.001	32.2	0.219
Others	2	467	Random	2.32(0.81 - 6.68)	0.118	82.5	0.017
Survival analysis							
Univariate	3	336	Fixed	2.99(1.96 - 4.58)	<0.001	34.7	0.216
Multivariate	3	624	Random	2.20(1.19 - 4.07)	0.012	67.2	0.047

R/R, relapsed/refractory; ISS, International Staging System; ASCT, autologous stem cell-transplanted; ROC, receiver operating characteristic; OS, overall survival; PNI, prognostic nutritional index; MM, multiple myeloma.

### PNI and PFS

**Figure 3:**
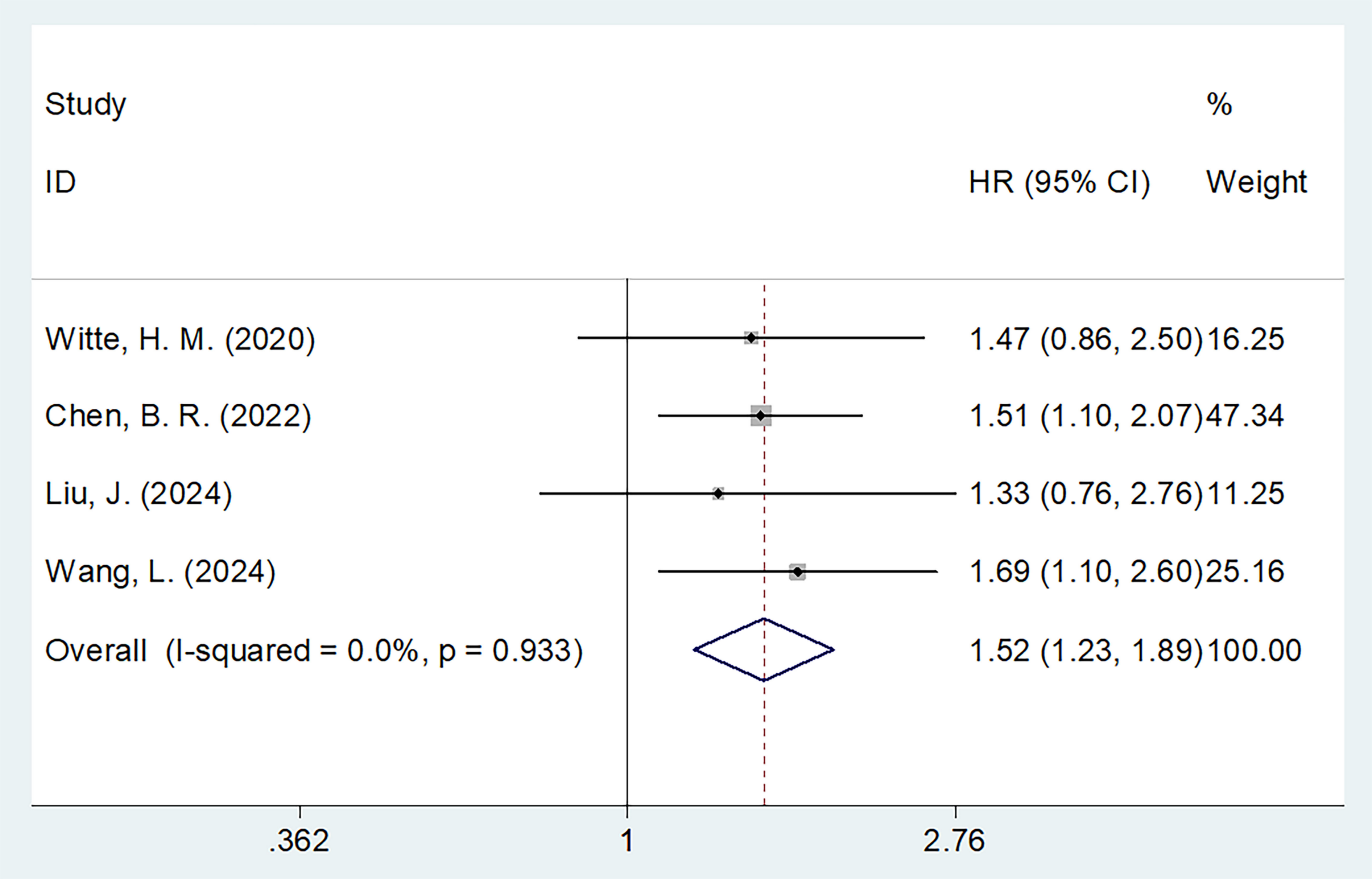
Forest plots of the association between PNI and PFS in patients with MM.

Four studies comprising 646 patients (15, 17, 20, 21) reported the relationship between PNI and PFS in MM. Because of non-significant heterogeneity (I^2^ = 0, p=0.933), we used the fixed-effects model. Consequently, decreased PNI markedly forecast unfavorable PFS of MM (HR = 1.52, 95% CI = 1.23-1.89, p<0.001; **Figure 3**; **Table 3**). In subgroup analyses, PNI significantly predicted PFS, despite the sample size, threshold, and threshold determination (**Table 3**). Additionally, decreased PNI was significantly associated with PFS in subgroups of Chinese studies (p<0.001), ISS stages I–III (p<0.001), patients treated with chemotherapy (p<0.001), and univariate survival analysis (p<0.001) (**Table 3**).

**Table 3:** Subgroup analysis of the prognostic role of PNI for PFS in patients with MM.

Subgroups	No. of studies	No. of patients	Effects model	HR (95%CI)	p	Heterogeneity I2(%) Ph	
Total	4	646	Fixed	1.52(1.23 - 1.89)	<0.001	0	0.933
Country							
Germany	1	224	–	1.47(0.86 - 2.50)	0.160	–	–
China	3	422	Fixed	1.54(1.21 - 1.94)	<0.001	0	0.815
Sample size							
≤160	2	261	Fixed	1.47(1.11 - 1.95)	0.007	0	0.720
>160	2	385	Fixed	1.60(1.14 - 2.24)	0.006	0	0.684
ISS stage							
I-III	3	545	Fixed	1.55(1.23 - 1.95)	<0.001	0	0.893
R/R	1	101	–	1.33(0.70 - 2.52)	0.391	–	–
Treatment							
ASCT	1	224	–	1.47(0.86 - 2.50)	0.160	–	–
Chemotherapy	3	422	Fixed	1.54(1.21 - 1.94)	<0.001	0	0.815
PNI cut-off value							
<45	2	261	Fixed	1.47(1.11 - 1.95)	0.007	0	0.720
≥45	2	385	Fixed	1.60(1.14 - 2.24)	0.006	0	0.684
Cut-off determination							
ROC curve	2	384	Fixed	1.57(1.10 - 2.24)	0.013	0	0.535
Others	2	262	Fixed	1.50(1.14 - 1.96)	0.003	0	0.927
Survival analysis							
Univariate	3	422	Fixed	1.54(1.21 - 1.94)	<0.001	0	0.815
Multivariate	1	224	–	1.47(0.86 - 2.50)	0.160	–	–

R/R, relapsed/refractory; ISS, International Staging System; ASCT, autologous stem cell-transplanted; ROC, receiver operating characteristic; PFS, progression-free survival; PNI, prognostic nutritional index; MM, multiple myeloma.

### Correlations of PNI with MM clinicopathological features

**Figure 4:**
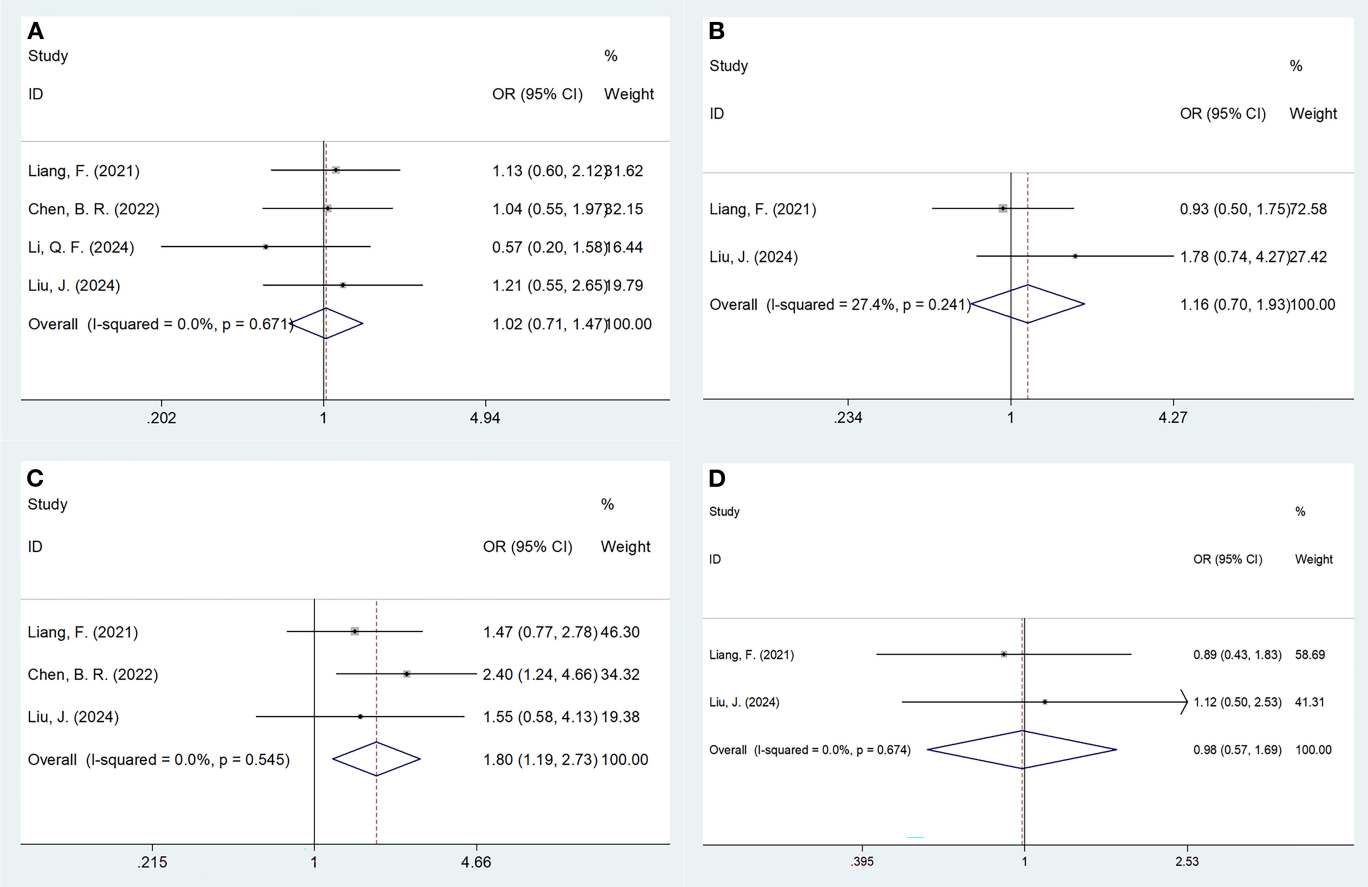
Meta-analyses of the association between PNI and clinicopathological features of MM. **(A)** Gender (male vs female); **(B)** Age (years) (>65 vs ≤65); **(C)** ISS stage (III vs I-II); and **(D)** LDH (U/L) (>220 vs ≤220).

Four studies comprising 492 patients (16, 17, 19, 20) reported connections between PNI and MM clinicopathological factors. The pooled data demonstrated that a low PNI was associated with ISS stage III (OR = 1.80, 95% CI = 1.19–2.73, p=0.005; **Figure 4**; **Table 4**). However, correlations between PNI and sex (OR = 1.02, 95% CI = 0.71–1.47, p=0.900), age (OR = 1.1, 95% CI = 0.70–1.93, p=0.558), and lactate dehydrogenase (OR = 0.98, 95% CI = 0.57–1.69, p=0.955) were not significant in MM (**Figure 4**; **Table 4**).

**Table 4:** The association between PNI and clinicopathological factors in patients with MM.

Factors	No. of studies	No. of patients	Effects model	OR (95%CI)	p	Heterogeneity I^2^(%) Ph	
Gender (male vs female)	4	492	Fixed	1.02(0.71 - 1.47)	0.900	0	0.671
Age (years) (>65 vs ≤65)	2	258	Fixed	1.16(0.70 - 1.93)	0.558	27.4	0.241
ISS stage (III vs I-II)	3	418	Fixed	1.80(1.19 - 2.73)	0.005	0	0.545
LDH (U/L) (>220 vs ≤220)	2	258	Fixed	0.98(0.57 - 1.69)	0.955	0	0.674

ISS, International Staging System; PNI, prognostic nutritional index; MM, multiple myeloma; LDH, lactate dehydrogenase.

### Sensitivity analysis

**Figure 5:**
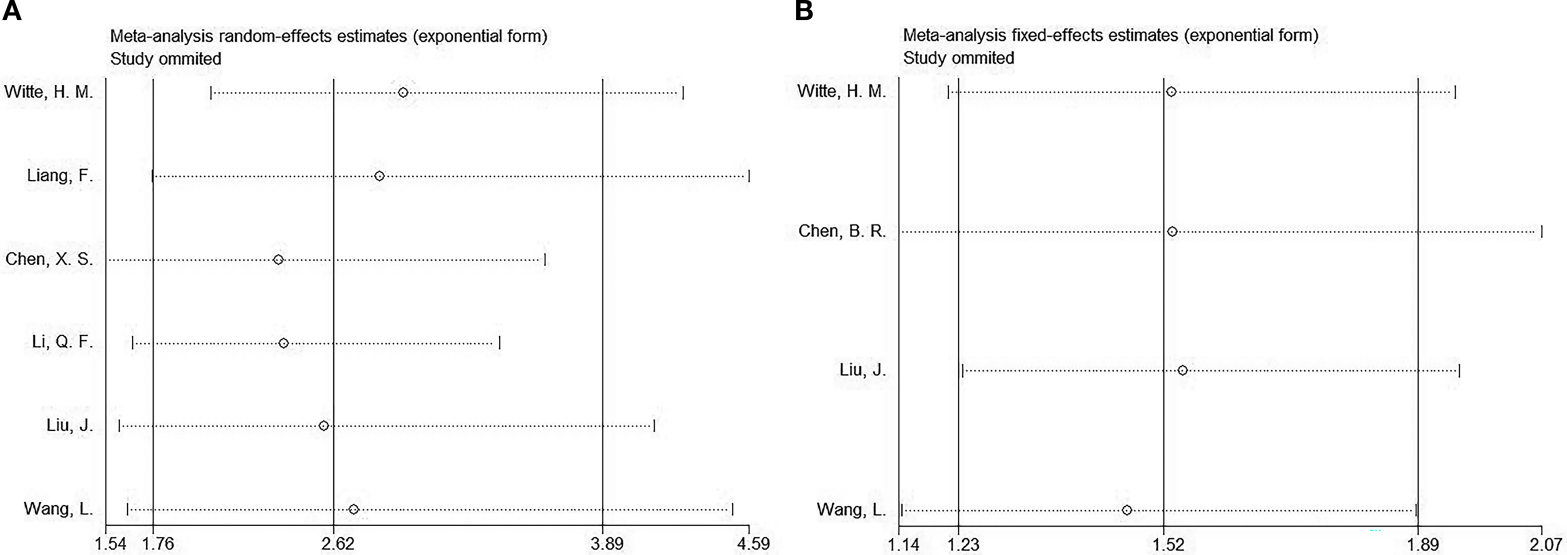
Sensitivity analysis. **(A)** OS and **(B)** PFS.

We performed a sensitivity analysis of the OS and PFS results to assess the effect of articles on the overall HR by removing them gradually. The combined HRs and 95% CIs showed no significant changes, indicating the robustness of the findings (**Figure 5**).

### Publication bias

**Figure 6:**
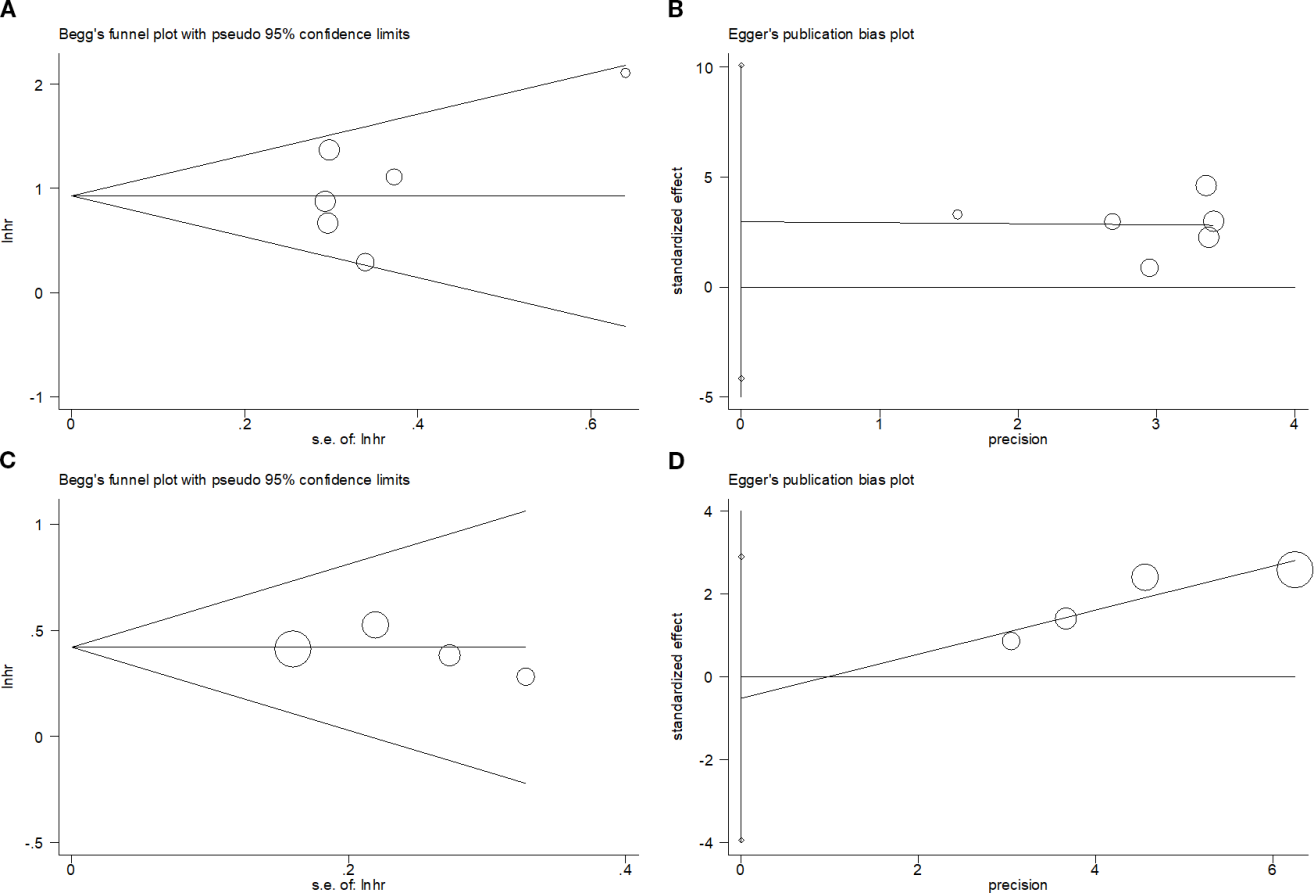
Publication bias test. **(A)** Begg’s test for OS, p=0.452; **(B)** Egger’s test for OS, p=0.311; **(C)** Begg’s test for PFS, p=0.308; and **(D)** Egger’s test for PFS, p=0.578.

This study performed Begg’s and Egger’s tests to evaluate potential publication bias, which did not detect a publication bias for OS (p=0.452/0.311 from Begg’s/Egger’s tests) or PFS (p=0.308/0.578 from Begg’s/Egger’s tests) (**Figure 6**).

## Discussion

The role of PNI in predicting MM prognosis has been widely explored; however, the findings remain conflicting. Therefore, we aggregated data from seven studies with 1120 patients (15–21). A lower PNI was significantly related to poor OS and dismal PFS in patients with MM. The significant prognostic role of PNI for OS and PFS remained unaffected by sample size and threshold. Moreover, a lower PNI correlated with ISS stage III in MM. Based on the publication bias test and sensitivity analysis, the findings were stable. Collectively, the PNI remarkably predicts the long- and short-term prognoses of MM. The present work provides initial meta-analysis evidence for the significance of PNI in MM prognosis forecasting.

PNI includes ALB and lymphocyte count, and a low PNI can be due to these two elements. The mechanisms by which the PNI predicts MM prognosis remain unclear and are explained below. Individuals with advanced cancer frequently experience nutritional depletion, and ALB is a marker of nutritional status. Serum ALB is essential for plasma osmotic pressure and nutrition and exerts an important effect on regulating body fluid distribution and acid-based balance (24). There is a relationship between diminished ALB levels and elevated inflammation and tumor progression (25). Furthermore, albumin, which is the predominant component of plasma protein, is an essential biomarker for evaluating nutritional status and associated with the comorbidity and prognosis of various cancers (26). Cancer treatments, such as chemotherapy or radiotherapy, may cause decreased appetite, leading to less protein consumption and disrupted synthesis (27). If tissue damage and inflammation occur, they can accelerate this catabolic process, thereby reducing plasma ALB levels (28). Conversely, through cytokine-related cytotoxicity, lymphocytes aid in cellular immunity by inhibiting cancer cell growth and migration (29). The lymphocyte count serves as a basic parameter for assessing immune function, and its reduction can be attributed to malnutrition and weakened cellular immune function (30). Studies indicate that lymphocytes have an essential effect on tumor immune surveillance, limiting cancer growth and spread by causing cytotoxic death and releasing cytokines that suppress tumors (31). Several studies have shown that lymphocytopenia is associated with an impaired anti-tumor immune response, and lymphocyte levels can be used as an indicator to predict treatment outcomes in patients with cancer (32, 33). Therefore, a low PNI indicates a lack of proper nutrition and immune function in patients with cancer, which may lead to more aggressive tumor behavior.

Notably, this meta-analysis showed that a lower PNI correlated with ISS stage III in MM. As part of the ISS for MM, serum ALB has been demonstrated to be the most consistent prognostic factor across virtually all research on MM (34). Therefore, reduced PNI, which is associated with lower ALB levels, may correspond with a higher ISS stage, such as stage II or III. The subgroup analysis revealed that PNI remained a significant prognostic marker for OS and PFS in patients with MM treated with chemotherapy, but not for ASCT (**Tables 2** and **3**). Because only one study treated patients with ASCT. The prognostic role of PNI for patients with MM receiving ASCT should be verified in more studies.

The R-ISS (Revised International Staging System) incorporates the variables from the original ISS, such as serum beta-2 microglobulin and serum albumin, and adds prognostic data from serum LDH and high-risk chromosomal abnormalities identified through interphase fluorescent *in situ* hybridization (iFISH) following CD138 plasma cell purification (35). In this meta-analysis, only one study (20) included provide the information on correlation between PNI and R-ISS in MM. This study reported that there was no significant association between PNI and R-ISS in MM (p=0.893). The sample size was limited; therefore, more studies should investigate the correlation between PNI and R-ISS in the future. Moreover, ASCT is utilized in the consolidation treatment for all myeloma patients, regardless of their ISS-R stage, and most are considered candidates for this treatment.

Current evidences show that there are some nutritional indexes for cancer prognostication, such as controlling nutritional status (CONUT) score, nutritional risk index (NRI), and body mass index (BMI) (11). There are some similarities and differences between these nutritional parameters and PNI. The similarities are as follows. First, they are all indexes reflecting the nutritional status of patients. Second, they all can be applied as prognostic indicators in cancer. The differences are as follows. PNI is calculated based on two elements. Therefore, PNI is easier to be computed compared with CONUT and NRI, which are calculated according to three elements.

PNI has been widely investigated for its significance in predicting cancer prognosis by mete-analysis (36–40). Tobing et al. suggested in their meta-analysis of 2,229 cases that a low PNI predicted shorter OS and PFS in prostate cancer (36). Zhang et al. suggested a significant relationship between a lower PNI and poorer OS and PFS among patients with endometrial cancer from their meta-analysis of 10 articles (37). In a meta-analysis comprising 23,756 cases, a low PNI was significantly associated with shorter OS, (RFS, and cancer-specific survival (CSS) in patients with gastric cancer undergoing gastrectomy (38). Based on a recent meta-analysis of 3,130 cases, low pretreatment PNI predicted unfavorable OS and disease-free survival (DFS) for oral cancer (39). Xue et al. suggested from a meta-analysis containing 15 articles that a lower pretreatment PNI level predicted unfavorable OS, PFS, CSS, and DFS in patients with renal cell carcinoma (40).

This study has certain limitations. First, most of the enrolled articles were from China; therefore, our findings are suitable for Chinese MM cases. No regional or language restrictions were applied in the literature search and infiltration. Second, the sample size is relatively small. This study enrolled only 1,120 cases. Third, the thresholds were non-uniform among the eligible articles. Fourth, the absence of information on the induction regimen, cycle count, dosage schedule, and post-induction treatments, such as transplant or maintenance therapy, complicates the acceptance of these results. Fifth, only one eligible study used ASCT. ASCT is currently the standard consolidation treatment for every myeloma patient following successful induction, typically after 4 cycles, and can be offered to patients up to 70 years old or even beyond. Six studies did not use ASCT in this meta-analysis. Moreover, it would be useful to analyze larger databases, such as SEER. Therefore, large-scale, cross-regional prospective investigations are necessary to verify the findings of this meta-analysis.

## Conclusion

In summary, a lower PNI evidently predicts unfavorable OS and PFS among MM cases and is significantly correlated with an advanced ISS stage of MM.

## Data Availability

The original contributions presented in the study are included in the article/**Supplementary Material**. Further inquiries can be directed to the corresponding author.
