# Supplementary material for: Prognostic and clinicopathological value of prognostic nutritional index in patients with multiple myeloma: a meta-analysis
Source: Front Oncol. 2025 Sep 19;15:1545096. doi: 10.3389/fonc.2025.1545096 (PMC12490979; doi:10.3389/fonc.2025.1545096)
Supplement: Supplementary file 1 [file Table1.docx]

Supplemental file 1 The detailed NOS score of each included study in this meta-analysis.

| Study | Year | Selection (0-4 points) | | | | Comparability  (0-2 points) | Outcome  (0-3 points) | | | Total score |
| --- | --- | --- | --- | --- | --- | --- | --- | --- | --- | --- |
|  |  | Representativeness of the exposed cohort | Selection of the non exposed cohort | Ascertainment of exposure | Demonstration that outcome of interest was not present at start of study | Comparability of cohorts on the basis of the design or analysis | Assessment of outcome | Was follow-up long enough for outcomes to occur | Adequacy of follow up of cohorts |  |
| Witte, H. M. | 2020 | ★ | ★ | ★ | ★ | ★★ | ★ | ★ | ★ | 9 |
| Liang, F. | 2021 | ★ | ★ | ★ | ★ | ★☆ | ★ | ★ | ★ | 8 |
| Chen, B. R. | 2022 | ★ | ★ | ★ | ★ | ★★ | ★ | ☆ | ★ | 8 |
| Chen, X. S. | 2023 | ★ | ★ | ☆ | ★ | ★★ | ★ | ★ | ★ | 8 |
| Li, Q. F. | 2024 | ★ | ★ | ☆ | ★ | ★☆ | ★ | ★ | ★ | 7 |
| Liu, J. | 2024 | ★ | ★ | ★ | ★ | ★☆ | ★ | ★ | ★ | 8 |
| Wang, L. | 2024 | ★ | ★ | ★ | ★ | ★☆ | ★ | ☆ | ★ | 7 |

NOS: Newcastle-Ottawa Scale; a ★ represents 1 point; a ☆ represents 0 point.
